# Differences found in patient profiles and incidence trends between migrants and native-born tuberculosis patients in Ireland: A cross-sectional analysis of national surveillance data, 2011-2021

**DOI:** 10.1016/j.ijregi.2025.100763

**Published:** 2025-09-15

**Authors:** Sarah Jackson, Zubair Kabir, Catherine Comiskey

**Affiliations:** 1 School of Nursing and Midwifery, Trinity College Dublin, The University of Dublin, Dublin, Ireland; 2 Health Protection Surveillance Centre, Health Service Executive, Dublin, Ireland; 3 School of Public Health, University College Cork, Cork, Ireland

**Keywords:** Tuberculosis, Epidemiology, Incidence, Public health, Emigration and immigration

## Abstract

**Objectives:**

Tuberculosis (TB) remains a global public health threat that was responsible for 1.3 million deaths in 2022 alone. Although Ireland is a low TB incidence country, with crude incidence rates (CIRs) in the Irish-born below six per 100,000 population since 2011, CIRs in the foreign-born population are up to 13 times higher. This study aims to inform TB prevention and care by analyzing the differences in the epidemiology of TB in native-born and foreign-born populations in Ireland.

**Methods:**

A cross-sectional analysis of all TB notifications reported to the Irish TB Surveillance System from 2011-2021 was performed. Temporal trends in CIRs were analyzed using negative-binomial regression. Independent variables selected with a *P*-value of <0.25 in univariable analysis were investigated in a multivariable logistic regression model comparing TB patient characteristics between migrants and Irish-born.

**Results:**

Of the 3364 TB patients, 48% were among migrants. Compared with Irish-born, migrants with TB were younger, had higher odds of living with HIV (odds ratio [OR] 3.8, confidence interval [CI] 1.99-7.73), extrapulmonary disease (OR 3.14, CI 2.09-4.79), infection with drug-resistant strains (OR 2.30, CI 1.37-4.01), and residence in congregate residential settings (OR 2.00, CI 1.34-3.05) with lower odds linkage to outbreaks (OR 0.16, CI 0.09-0.28). Recently arrived migrants with TB had higher proportions of international protection applicants and refugees, pulmonary disease, and people living with HIV. Between 2011 and 2021, a significantly declining temporal trend was present for migrants (incidence rate ratio [IRR] 0.96, CI 0.95-0.98), Irish-born (IRR 0.89; CI 0.86-0.92), and total TB patients (IRR 0.95, CI 0.94-0.96). Between 2017 and 2021, a significantly declining temporal trend was still present in Irish-born (IRR 0.76, CI 0.69-0.83) and total patients (IRR 0.91, CI 0.88-0.95), but the trend was no longer significant among migrants with TB (IRR 0.96, CI 0.91-1.01).

**Conclusions:**

A heightened awareness of extrapulmonary TB within health systems is needed, given the high levels observed among migrants with TB. The pace of TB decline among migrants is no longer significantly declining in the final years of this study period, making TB elimination targets more difficult to achieve. Differences in the epidemiology of TB reported by this study can be used to inform and enhance future TB service provision and promote migrant health.

## Highlights

• Tuberculosis (TB) incidence rates no longer significantly declining in migrants for the latter half of the study period.
• Key epidemiological differences were found between migrants and Irish-born cases.
• Migrants with TB were less likely to have pulmonary TB and be linked to outbreaks.
• Migrants had higher odds of drug resistance and living with HIV than Irish cases.
• Recently arrived migrants had a higher level of pulmonary disease and living with HIV.

## Graphical Abstract

**Figure:**
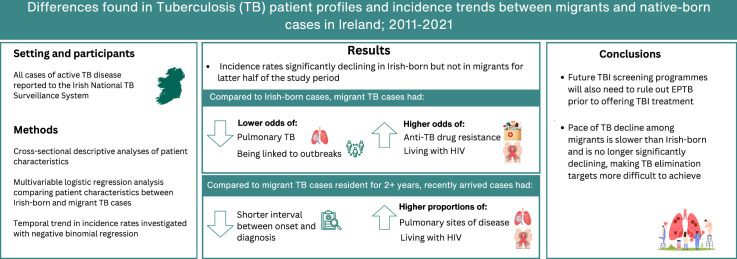

## Introduction

Tuberculosis (TB) remains a global public health threat that was responsible for 1.3 million deaths in 2022 alone [1]. Ireland has experienced a steady decline in TB since the 1950s, when crude incidence rates (CIRs) were over 220 per 100,000 population, to a CIR of <5 per 100,000 during 2023, becoming a low TB incidence country in 2010 [2]. Although CIRs in the Irish-born remained below six per 100,000 population since 2011, CIRs in the foreign-born population have been up to 13 times higher [2].

Migrants are a very diverse population group, and consequently, their TB risk is influenced by many factors. Background risk of infection in the country of origin is a key component; however, the process of migration itself can alter the risk of acquiring a new infection or developing TB disease. Although migrant populations often comprise individuals who are young, financially stable, and healthy enough to travel, sub-populations who are forced to travel via hazardous land and sea migration routes (such as International Protection Applicants and Refugees) are at increased risk due to the overcrowding, food insecurity, and stressful conditions that can occur [3,4]. After arrival in the destination country, the risk of TB may be further influenced by migrant demography and health status, along with structural and social determinants such as the presence of screening programs, health system accessibility, and socioeconomic profile [[5], [6], [7]].

International migration is increasing globally, with the European region experiencing some of the steepest increases in recent years, from 50 million international migrants in 1990 to 87 million international migrants in 2020 [8]. Similarly, Ireland has also experienced a steady increase in the proportion of the population that was born abroad, from 10% in 2002 to 20% in 2022 [9,10]. Given these increasing global migration trends, stimulated by rising environmental and political instability, migrants remain a key population for TB prevention and care activities. For this reason, an enhanced understanding of the epidemiologic patterns among native-born and foreign-born TB patients is required.

This study aims to inform future TB prevention and care activities by analyzing the differences in the epidemiology of TB in native-born and foreign-born populations within the Irish context and by providing an in-depth analysis of patient profiles of migrants with TB.

## Methods

### Study population

We performed a cross-sectional secondary data analysis of all TB disease notifications reported to the Irish National TB Surveillance System and notified to the Computerised Infectious Disease Reporting (CIDR) system between 2011 and 2021 [8].

### Definitions

The European Union Commission TB surveillance case definition for TB disease was used [11]. TB disease (formerly known as “active TB”) was defined as cases meeting the clinical criteria of signs, symptoms, and/or radiological findings consistent with TB disease in any site and a clinician’s decision to treat the person with a full course of anti-TB therapy, or a case discovered post-mortem with pathological findings consistent with TB disease that would have indicated anti-TB antibiotic treatment had the patient been diagnosed before dying. No restrictions on case classification were used; possible, probable, and confirmed case classifications were included. Cases were classified as follows:

• Confirmed case – A person meeting the clinical criteria and at least one of the following two:
  ▪ Isolation of *Mycobacterium tuberculosis* complex* (excluding *Mycobacterium bovis* BCG) from a clinical specimen OR
  ▪ Detection of *M. tuberculosis* nucleic acid in a clinical specimen AND
  ▪ Positive microscopy for acid-fast bacilli or equivalent fluorescent staining bacilli on light microscopy.
• Probable case – A person meeting the clinical criteria and at least one of the following three:
  ▪ Microscopy positive for acid-fast bacilli or equivalent fluorescent staining bacilli on light microscopy OR
  ▪ Detection of *M. tuberculosis* nucleic acid in a clinical specimen OR
  ▪ Histological appearance of granulomata.
• Possible case: A person meeting the clinical criteria without laboratory confirmation.

Migrants with TB were defined as patients who were born outside Ireland but notified with TB in Ireland. Resistance to TB drugs was defined as resistance to one or more of the following drugs: isoniazid, rifampicin, ethambutol, pyrazinamide, streptomycin, any second-line injectable (e.g., amikacin, kanamycin), or any fluoroquinolone (e.g., levofloxacin, moxifloxacin). Multidrug resistance (MDR) was defined as resistance to both isoniazid and rifampicin, and extensively drug-resistant (XDR) TB was defined using the pre-2021 World Health Organization (WHO) definition of resistance to rifampicin and any fluoroquinolone and a second-line injectable.

### Data analysis

Chi-squared test for trend in proportions, Kruskal-Wallis, or Wilcoxon rank sum tests were used to test associations between independent variables and categorical outcomes. CIRs and age-specific incidence rates per 100,000 population were calculated using 2016 census population denominators stratified by country of birth and age. Temporal trends in CIRs were analyzed using negative-binomial regression and incidence rate ratios (IRRs). Temporal trends were assessed for total patients, migrant patients, and Irish-born patients for both the full study period (2011-2021) and the latter half (2017-2021).

Independent variables selected based on clinical relevance and literature review, with a *P*-value of ≤0.25 in univariable analysis, were investigated in a multivariable logistic regression model comparing patient characteristics of migrants with TB compared with Irish-born with TB. We assessed the potential effect of excluding patients with missing data from the model by comparing the distribution of each of the 11 selected independent variables with the dependent variable within the complete cases sample and within the total sample. The distributions were broadly similar between the two samples. Independent variables were assessed for co-linearity using variable inflation factor analysis and removed if the variable inflation factor was >9.0. Variables with the highest Wald *P*-value and lowest likelihood ratio test statistic were removed one at a time through backwards stepwise elimination until all variables had a Wald *P*-value of <0.05. Model fit was assessed using Hosmer-Lemeshow’s test, and models were compared using the Akaike information criterion weights.

The mean TB incidence estimate per 100,000 population for each migrant birth country for the study period was calculated using the annual incidence estimates published by the WHO [12]. Birth countries were then classified as one of the following incidence categories: low = 0.0-9.9, medium = 10.0-39.9, high = 40.0-99.9, and very high ≥100.

Time between arrival in Ireland and notification as a TB patient was estimated by subtracting the arrival year from the notification year and categorized as follows: <2 years, 2-4 years, 5-9 years, and ≥10 years.

Data were analyzed using MS Excel, Stata 14, and R software [13,14].

### Protection of human subjects

Ethical approval was received from the research ethics committee in the School of Nursing and Midwifery, Trinity College Dublin. Informed consent was not sought from the patients in this study as the legal basis for processing surveillance data in Ireland is not consent but based on General Data Protection Regulations^1^, Articles 6(1)(c) and 6(1)(e); Articles 9(2)(i) and 9(2)(j) and the Infectious Disease Regulations^2^. Furthermore, as this was a secondary data analysis of anonymized data, there was no requirement for informed consent under the Health Research Regulations Act 2018^3^. All data processing was compliant with the General Data Protection Regulations.

## Results

### Epidemiology of tuberculosis among migrant patients compared with Irish-born

Of the 3364 TB patients notified between 2011 and 2021, 48% (n = 1605) were among migrants, 47% (n = 1593) were among Irish-born, and 5% (n = 166) did not have country of birth reported. Overall, 5% (n = 153) of total patients were reported as being an International Protection Applicant or Refugee (IPARs). Table 1 summarizes the overall distribution of patient characteristics alongside results of univariable and multivariable logistic regression analysis comparing characteristics of migrants with TB to TB among Irish-born.

**Table 1:** TB patient characteristics by migrant status and logistic regression analysis results, Ireland 2011-2021.

Characteristic^a^	Missing	Overall N = 3198	Non-migrant N = 1593	Migrant N = 1605	Univariable		Multivariable (N = 825)	
					Crude OR (95% CI)	*P*-value	Adjusted OR (95% CI)	*P*-value
**Median age (interquartile range)**	4 (0.1%)	40.0 (28.0)	53.0 (33.0)	34.0 (16.0)	0.95 (0.95-0.96)	**<0.001**	0.97 (0.95-0.98)	**<0.001**
**Sex**	4 (0.1%)					**<0.001**		
*Female*		1276 (40%)	588 (37%)	688 (43%)	—			
*Male*		1918 (60%)	1005 (63%)	913 (57%)	0.78 (0.67-0.89)			
**Geographical area**	0 (0%)					**<0.001**		**0.037**
*East*		1387 (43%)	563 (35%)	824 (51%)	—		—	
*Midlands*		155 (4.8%)	69 (4.3%)	86 (5.4%)	0.85 (0.61-1.19)		3.26 (1.24-9.69)	
*Midwest*		196 (6.1%)	104 (6.5%)	92 (5.7%)	0.60 (0.45-0.82)		1.10 (0.63-1.92)	
*North East*		228 (7.1%)	115 (7.2%)	113 (7.0%)	0.67 (0.51-0.89)		1.45 (0.57-4.05)	
*North West*		115 (3.6%)	77 (4.8%)	38 (2.4%)	0.34 (0.22-0.50)		0.62 (0.27-1.47)	
*South*		610 (19%)	396 (25%)	214 (13%)	0.37 (0.30-0.45)		0.65 (0.41-1.03)	
*South East*		262 (8.2%)	146 (9.2%)	116 (7.2%)	0.54 (0.42-0.71)		0.76 (0.46-1.28)	
*West*		245 (7.7%)	123 (7.7%)	122 (7.6%)	0.68 (0.52-0.89)		1.16 (0.58-2.37)	
**Employment status**	371 (12%)					**<0.001**		**0.024**
*Paid employment*		1041 (37%)	399 (28%)	642 (45%)	—		—	
*Unemployed*		929 (33%)	428 (30%)	501 (35%)	0.73 (0.61-0.87)		0.82 (0.56-1.19)	
*Retired*		443 (16%)	403 (29%)	40 (2.8%)	0.06 (0.04-0.09)		0.27 (0.12-0.60)	
*Student/Child*		329 (12%)	135 (9.6%)	194 (14%)	0.89 (0.69-1.15)		0.86 (0.45-1.70)	
*Other*		85 (3.0%)	46 (3.3%)	39 (2.8%)	0.53 (0.34-0.82)		0.81 (0.35-1.90)	
**Current housing type**	343 (11%)					**<0.001**		
*Private house*		2646 (93%)	1352 (94%)	1294 (92%)	—			
*Congregate residential setting*	105 (3.7%)	36 (2.5%)	69 (4.9%)	2.00 (1.34-3.05)				
*Homeless*		27 (0.9%)	16 (1.1%)	11 (0.8%)	0.72 (0.32-1.54)			
*Prison*		22 (0.8%)	9 (0.6%)	13 (0.9%)	1.51 (0.65-3.67)			
*Residential care facility*		20 (0.7%)	18 (1.2%)	2 (0.1%)	0.12 (0.02-0.40)			
*Other housing*		35 (1.2%)	14 (1.0%)	21 (1.5%)	1.57 (0.80-3.16)			
**Disease site**	36 (1.1%)					**<0.001**		**<0.001**
*Pulmonary*		2140 (68%)	1223 (78%)	917 (58%)	—		—	
*Extrapulmonary*		1022 (32%)	353 (22%)	669 (42%)	2.53 (2.17-2.95)		3.14 (2.09-4.79)	
**Outbreak associated**	0 (0%)					**<0.001**		**<0.001**
*Not linked to outbreak*		2875 (90%)	1345 (84%)	1530 (95%)	—		—	
*Outbreak associated*		323 (10%)	248 (16%)	75 (4.7%)	0.27 (0.20-0.35)		0.16 (0.09-0.28)	
**People living with HIV**	1804 (56%)					**<0.001**		**<0.001**
*Negative*		1266 (91%)	554 (96%)	712 (87%)	—		—	
*Positive*		128 (9.2%)	22 (3.8%)	106 (13%)	3.75 (2.38-6.16)		3.80 (1.99-7.73)	
**First-line drug resistance**	838 (26%)					**<0.001**		**0.001**
*Sensitive*		2065 (88%)	1046 (93%)	1019 (83%)	—		—	
*Resistant*		295 (13%)	82 (7.3%)	213 (17%)	2.67 (2.05-3.51)		2.30 (1.37-4.01)	
**Multi/extensively drug-resistant-TB**	838 (26%)					**<0.001**		
*No*		2315 (98%)	1126 (100%)	1189 (97%)	—			
*Yes*		45 (1.9%)	2 (0.2%)	43 (3.5%)	20.4 (6.26-125)			
**Previous TB screening in Ireland**	887 (28%)					**<0.001**		
*No*		1953 (85%)	908 (80%)	1045 (89%)	—			
*Yes*		358 (15%)	224 (20%)	134 (11%)	0.52 (0.41-0.65)			
**Previous TB diagnosis**	801 (25%)					**0.086**		
*No*		2188 (91%)	1089 (90%)	1099 (92%)	—			
*Yes*		209 (8.7%)	117 (9.7%)	92 (7.7%)	0.78 (0.58-1.04)			
**Patient type**	362 (11%)					**0.004**		
*Hospital inpatient*		1658 (58%)	876 (61%)	782 (56%)	—			
*Hospital outpatient*		914 (32%)	425 (29%)	489 (35%)	1.29 (1.10-1.52)			
*Other*		264 (9.3%)	145 (10%)	119 (8.6%)	0.92 (0.71-1.19)			
**Diabetes**	1862 (58%)					>0.99		
*No*		1204 (90%)	648 (90%)	556 (90%)	—			
*Yes*		132 (9.9%)	71 (9.9%)	61 (9.9%)	1.00 (0.70-1.43)			
**Immunosuppression**	1841 (58%)					**0.033**		
*No*		1067 (79%)	569 (76%)	498 (81%)	—			
*Yes*		290 (21%)	175 (24%)	115 (19%)	0.75 (0.58-0.98)			
**Substance use**	1881 (59%)					**<0.001**		
*No*		945 (72%)	475 (62%)	470 (86%)	—			
*Yes*		372 (28%)	297 (38%)	75 (14%)	0.26 (0.19-0.34)			

CI, confidence interval; OR, odds ratio; TB, tuberculosis.

a “Congregate residential setting” includes those reported as being resident in the following types of accommodation at the time of their notification with TB: Bed and Breakfast/Hotel, Hostel, Institution, State Provided Migrant Accommodation.

#### Age and sex

Median ages were significantly younger among migrants with TB compared with Irish-born and among IPARs compared with non-IPAR migrants (31 vs 34 years, respectively). Cumulative age-specific incidence rates were higher among migrants with TB in all age groups (Figure A1). Male-to-female ratio was 1.3 among migrants with TB and 1.7 among Irish-born.

#### Clinical features

Lower proportions of pulmonary TB (PTB) were observed among migrants with TB (57.9%) compared with Irish-born (77.6%). Compared with Irish-born, migrants with TB also had higher odds of having normal chest X-ray results (odds ratio [OR] 3.13, confidence interval [CI] 2.45-4.02) and computerized tomography thorax scan results (OR 3.62, CI 1.92-7.26). A significantly shorter median interval between onset and diagnosis was observed among migrants with TB (63 days, range 0-3294) compared with Irish-born (75 days, range 0-2111) (*P* = 0.001).

#### Drug resistance

Drug susceptibility testing data were available for 73.8% (n = 2360) of the total patients. Infection with a drug-resistant strain was reported in 17.3% of migrants with TB and 7.3% of Irish-born. Forty patients with MDR-TB were migrants (90.9%), and all three patients with XDR-TB were migrants. Of the 47 patients with M/XDR-TB, 79.5% (n = 35) had PTB. Infection with non-MDR poly-resistant strains was reported in 40 patients (0.2%) from 17 countries of origin; seven (17.5%) of these were among Irish-born.

#### Multivariable logistic regression analysis results

After backwards stepwise elimination, seven independent variables remained as significant predictors of TB among migrants in the final model (Table 1). Compared with Irish-born, migrants with TB were younger, with a higher adjusted odds of living with HIV (OR 3.8, CI 1.99-7.73), extrapulmonary disease (OR 3.14, CI 2.09-4.79), infection with drug-resistant strains (OR 2.30, CI 1.37-4.01), and residence in the Midlands area of Ireland (OR 3.26, CI 1.24-9.69), but lower odds of being associated with an outbreak (OR 0.16, CI 0.09-0.28).

All metrics of the model assumptions were acceptable, with 83% of binned residuals within error bounds. Variable inflation factor analysis indicated that levels of co-linearity were low. Hosmer-Lemeshow’s test found no evidence of poor model fit (statistic 9.5, *P* = 0.304).

#### Temporal trends

**Figure 1:**
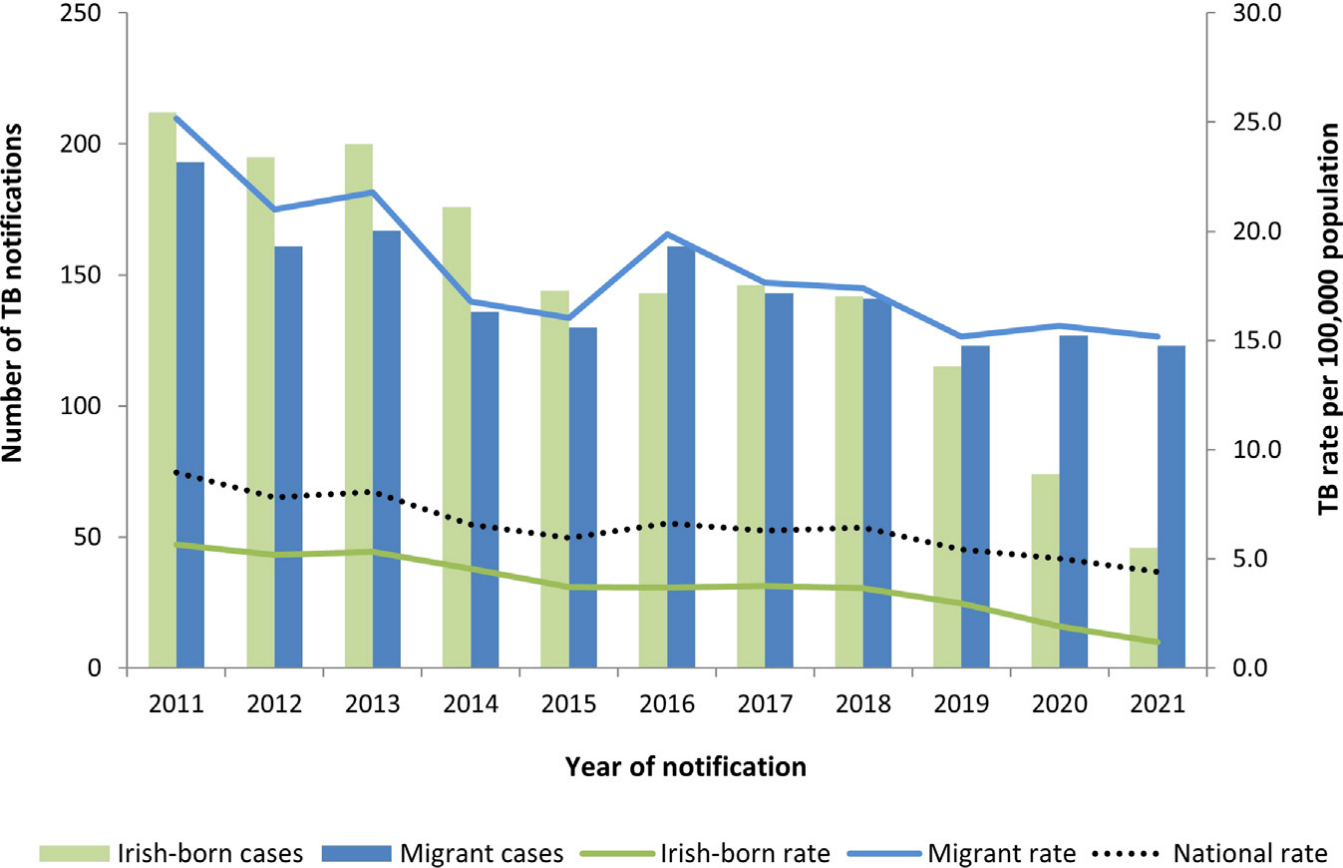
Annual number of TB patients and crude incidence rate per 100,000 population by migrant status.

Between 2011 and 2021, a significantly declining temporal trend was present in the annual CIRs per 100,000 population for all three patient cohorts analyzed: migrants with TB, Irish-born TB, and total TB patients. Between 2011 and 2021, annual CIRs declined from 9.0 to 4.4 for total patients (IRR 0.95, CI 0.94-0.96), from 25.2 to 15.2 among migrants with TB (IRR 0.96, CI 0.95-0.98), and from 5.6 to 1.2 among Irish-born (IRR 0.89, CI 0.86-0.92). Between 2017 and 2021, a significantly declining temporal trend was still present in Irish-born (IRR 0.76, CI 0.69-0.83) and total patients (IRR 0.91, CI 0.88-0.95), but the trend was no longer significant among migrants with TB (IRR 0.96, CI 0.91-1.01) (Figure 1).

TB, tuberculosis.

### Epidemiology of migrants with tuberculosis

Migrants with TB originated from 98 countries. The majority of migrants with TB (68.5%) originated from countries classified as very high TB incidence (CIR ≥100/100,000 population) according to the mean WHO incidence estimate for the study period. Annual CIRs by TB incidence category are illustrated in Figure A2.

Patient characteristics of migrants with TB differed according to the incidence level in the origin country (Table A1). Migrants with TB originating from very high-incidence countries had the highest proportions of people living with HIV (PLWH) and with previous TB screening in Ireland, and the lowest proportions with PTB and being linked to an outbreak. A significant trend in the proportion of IPARs was observed in relation to the increasing incidence category.

**Figure 2:**
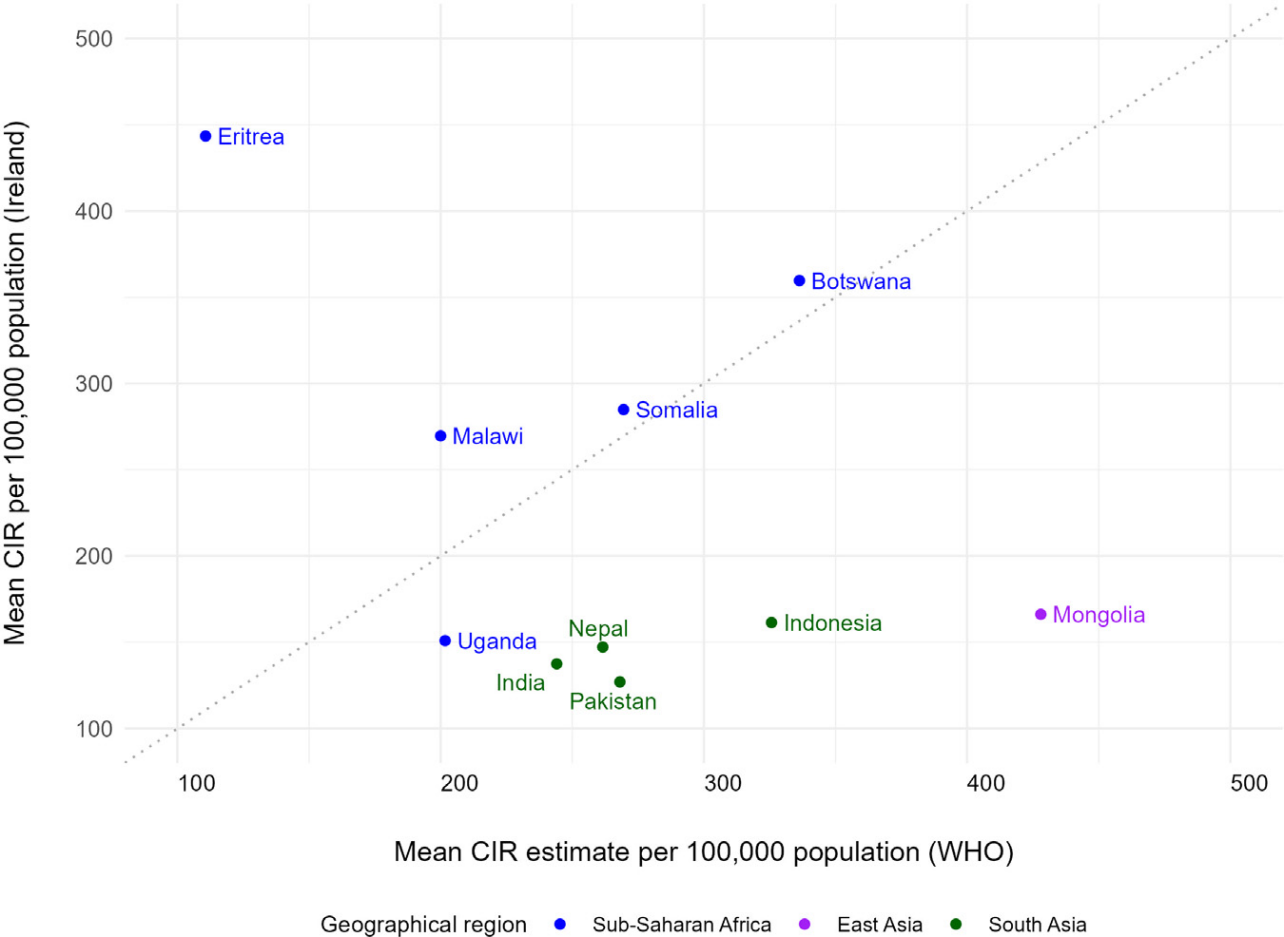
Origin countries with 10 highest mean crude incidence rates compared with the WHO incidence estimates, Ireland, 2011-2021.

CIR, crude incidence rate; WHO, World Health Organization.

The top 10 birth countries of migrants with TB differed when ranked by patient numbers vs by the mean annual CIR in Ireland, except for India, Pakistan, and Somalia, which were ranked by both metrics. Birth countries with the highest number of migrants with TB comprised six very high TB incidence countries, two high incidence and two medium incidence countries. Birth countries with the highest TB CIRs in this study were all classified as very high TB incidence countries according to the mean WHO CIR estimates. Table 2 displays the mean CIR and the percentage of migrants with TB originating from each of the birth countries for the study period in the top 10 category. CIRs for migrants with TB from Eritrea, Botswana, Malawi, and Somalia were higher in Ireland than the mean CIR estimates reported by the WHO in these countries (Figure 2). The proportion of IPARs was higher among patients from birth countries with the highest CIRs (9.8%) compared with the remaining countries (5.1%), with the highest proportions of IPARs found in migrants from Eritrea (75.0%), Somalia (27.7%), Uganda (12.5%), and Malawi (12.0%).

**Table 2:** Migrant source countries with top 10 highest percentage of patients or CIR between 2011 and 2021, Ireland.

Country of birth	Mean CIR	Median CIR	Mean WHO estimate	Ranking in Ireland	WHO Mean CIR category	Years with patients	Total patients	Percentage of migrant with tuberculosis
Eritrea	443.5	609.8	110.7	Top 10 CIR	Very high	7	8	0.5
Botswana	359.7	359.7	336.3	Top 10 CIR	Very high	6	11	0.7
Somalia	284.8	333.3	269.5	Top 10 CIR & number	Very high	10	47	2.9
Malawi	269.6	237.2	200.0	Top 10 CIR	Very high	8	25	1.6
Mongolia	166.2	261.1	428.0	Top 10 CIR	Very high	6	7	0.4
Indonesia	161.4	0.0	325.7	Top 10 CIR	Very high	5	6	0.4
Uganda	150.9	207.5	201.7	Top 10 CIR	Very high	6	8	0.5
Nepal	147.2	124.5	261.6	Top 10 CIR	Very high	8	13	0.8
India	137.4	133.5	244.1	Top 10 CIR & number	Very high	11	317	19.8
Pakistan	126.9	100.8	268.1	Top 10 CIR & number	Very high	11	180	11.2
Philippines	83.3	81.5	555.6	Top 10 number	Very high	11	135	8.4
South Africa	73.1	61.8	858.0	Top 10 number	Very high	11	65	4.0
Nigeria	36.8	30.2	219.0	Top 10 number	Very high	11	67	4.2
Romania	35.2	41.8	73.8	Top 10 number	High	11	111	6.9
Lithuania	12.3	12.0	48.9	Top 10 number	High	11	45	2.8
Poland	4.7	4.3	17.2	Top 10 number	Medium	11	59	3.7
United Kingdom	1.5	1.1	10.3	Top 10 number	Medium	11	47	2.9

CIR, crude incidence rate; WHO, World Health Organization.

#### Interval between arrival and notification

The year of arrival in Ireland was reported for 66.7% of migrants with TB. The median interval between arrival in Ireland and notification with TB was 5 years (mean = 7 years) and ranged from 0 to 59 years with years of arrival spanning from 1954 to 2021. The median interval was shorter between 2019 and 2021 (3 years). Migrants with TB who were diagnosed <2 years after arrival were significantly younger and had higher proportions of IPARs, PLWH, and PTB. The proportions of migrants with TB from very high-incidence countries decreased in line with length of stay in Ireland (72-65%). Table 3 summarizes the patient characteristics of migrants with TB according to the interval between their arrival in Ireland and notification with TB.

**Table 3:** Patient characteristics of migrants with TB by interval between arrival and notification with TB, Ireland.

Characteristic N (%)	Overall N = 1071	Interval between arrival in Ireland and TB diagnosis				*P*-value^a^
		0-1 N = 239	2-4 N = 295	5-9 N = 239	10+ N = 298	
**Median age (IQR)**	34.0 (15.0)	31.0 (12.0)	30.0 (11.0)	34.0 (12.0)	42.0 (16.0)	**<0.001**
*(% missing)*	0.2	0	0	0.4	0.3	
**Sex**						0.33
*Female*	473 (44%)	104 (44%)	120 (41%)	116 (49%)	133 (45%)	
*Male*	596 (56%)	135 (56%)	175 (59%)	123 (51%)	163 (55%)	
*(% missing)*	0.2	0	0	0	0.7	
**International protection applicant**						**<0.001**
*No*	799 (86%)	164 (77%)	223 (85%)	174 (89%)	238 (93%)	
*Yes*	127 (14%)	49 (23%)	38 (15%)	22 (11%)	18 (7.0%)	
*(% missing)*	14	11	12	18	14	
**WHO TB incidence category**						**0.016**
*Low*	20 (1.9%)	3 (1.3%)	1 (0.3%)	6 (2.5%)	10 (3.4%)	
*Medium*	110 (10%)	15 (6.3%)	23 (7.9%)	30 (13%)	42 (14%)	
*High*	184 (17%)	49 (21%)	50 (17%)	34 (14%)	51 (17%)	
*Very high*	749 (70%)	172 (72%)	217 (75%)	169 (71%)	191 (65%)	
*(% missing)*	0.7	0	1.4	0	1.3	
**Previous TB screening in Ireland**						**0.006**
*No*	814 (88%)	202 (94%)	226 (89%)	172 (84%)	214 (85%)	
*Yes*	113 (12%)	13 (6.0%)	29 (11%)	32 (16%)	39 (15%)	
*(% missing)*	13	10	14	15	15	
**Previous TB diagnosis**						0.67
*No*	839 (93%)	192 (92%)	225 (93%)	192 (95%)	230 (92%)	
*Yes*	65 (7.2%)	16 (7.7%)	17 (7.0%)	11 (5.4%)	21 (8.4%)	
*(% missing)*	16	13	18	15	16	
**Disease site**						**0.007**
*Pulmonary*	626 (59%)	163 (68%)	167 (57%)	129 (54%)	167 (56%)	
*Extrapulmonary*	443 (41%)	76 (32%)	127 (43%)	110 (46%)	130 (44%)	
*(% missing)*	0.2	0	0.3	0	0.3	
**Months between onset and diagnosis, median (IQR)**	2.0 (3.2)	1.7 (2.2)	2.1 (3.0)	2.0 (3.7)	2.6 (3.7)	**0.014**
*(% missing)*	32	33	30	33	31	
**Outbreak associated**						**0.034**
*Not linked to outbreak*	1,026 (96%)	232 (97%)	274 (93%)	232 (97%)	288 (97%)	
*Outbreak associated*	45 (4.2%)	7 (2.9%)	21 (7.1%)	7 (2.9%)	10 (3.4%)	
*(% missing)*	0	0	0	0	0	
**People living with HIV**						**0.004**
*Negative*	542 (89%)	103 (80%)	154 (91%)	120 (92%)	165 (90%)	
*Positive*	69 (11%)	26 (20%)	15 (8.9%)	10 (7.7%)	18 (9.8%)	
*(% missing)*	43	46	43	46	39	
**First-line drug resistance**						0.59
*Sensitive*	697 (81%)	158 (81%)	196 (79%)	165 (84%)	178 (81%)	
*Resistant*	160 (19%)	36 (19%)	52 (21%)	31 (16%)	41 (19%)	
*(% missing)*	20	19	16	18	27	
**Multi/extensively drug-resistant-TB**						0.39
*No*	826 (96%)	187 (96%)	235 (95%)	191 (97%)	213 (97%)	
*Yes*	31 (3.6%)	7 (3.6%)	13 (5.2%)	5 (2.6%)	6 (2.7%)	
*(% missing)*	20	19	16	18	27	
**Employment status**						**<0.001**
*Paid employment*	479 (46%)	76 (33%)	136 (47%)	115 (49%)	152 (52%)	
*Unemployed*	365 (35%)	92 (40%)	89 (31%)	81 (35%)	103 (35%)	
*Retired*	26 (2.5%)	9 (3.9%)	4 (1.4%)	2 (0.9%)	11 (3.8%)	
*Student/Child*	147 (14%)	44 (19%)	50 (17%)	32 (14%)	21 (7.2%)	
*Other*	29 (2.8%)	10 (4.3%)	10 (3.5%)	4 (1.7%)	5 (1.7%)	
*(% missing)*	2.3	3.3	2.0	2.1	2.0	
**Current housing type**						**<0.001**
*Private house*	952 (92%)	183 (79%)	271 (93%)	218 (94%)	280 (98%)	
*Congregate residential / care setting*	60 (5.8%)	35 (15%)	15 (5.2%)	8 (3.4%)	2 (0.7%)	
*Prison / homeless*	10 (1.0%)	2 (0.9%)	2 (0.7%)	4 (1.7%)	2 (0.7%)	
*Other housing*	16 (1.5%)	11 (4.8%)	2 (0.7%)	2 (0.9%)	1 (0.4%)	
*(% missing)*	3.1	3.3	1.7	2.9	4.4	

IQR, interquartile range; TB, tuberculosis; WHO, World Health Organization.

a Kruskal-Wallis rank sum test; Pearson’s chi-squared test.

## Discussion

This study reveals key clinical and epidemiological differences in the patient profile of migrants with TB compared with Irish-born and within the migrant subgroups studied. Compared with Irish-born, migrants with TB were younger, with higher odds of living with HIV, extrapulmonary TB (EPTB), infection with a drug-resistant strain, and residence in a congregate setting, with lower odds of being outbreak associated. Although a highly diverse range of origin countries was observed in this study, the majority of migrants with TB (69%) originated from very high TB incidence countries. This contrasts with the Irish population denominator, where 14% of migrants originated from high TB incidence countries [10].

Similar to studies in Europe and the United States [[15], [16], [17]], EPTB was more common among migrants, with three times higher adjusted odds compared with Irish-born. Accordingly, migrants with TB were almost twice as likely to have a normal computerized tomography thorax scan result and a normal chest X-ray result. Currently, there is no systematic TB screening program in Ireland, but thoracic TB symptom screening is offered mainly to international protection applicants (IPAs) and new-entrant health care professionals from high TB incidence countries [18]. Results from this study suggest that such screening could miss up to 40% of migrants with TB and may need to be supplemented by targeting high-risk migrant groups for TB infection (TBI) screening to support TB elimination in Ireland [19]. This finding supports the development of a new-entrant immigrant TBI management program as one of the key actions in Ireland’s Tuberculosis Strategy [20]. Proportions of EPTB increased in tandem with incidence in the origin country and, similar to European studies, were the highest among patients originating from South Asia [15], [16], [21]. In keeping with the lower levels of PTB, migrants with TB had lower odds of being associated with an outbreak, which is supported by findings in the literature that migrants with TB tend to contribute less to transmission [[22], [23], [24]].

This study found migrants with TB had almost four times higher adjusted odds of living with HIV than Irish-born, higher than reported by a large-scale European study, which found the proportions of PLWH among migrants with TB were twice as high compared with native-born TB [17]. Birth countries with the highest proportions of PLWH in this study were mainly from Sub-Saharan Africa, reflecting the co-epidemic of TB-HIV in this region [25]. Despite a strong recommendation to offer HIV testing to all TB patients [26], only 42% had their HIV status reported. Levels of completeness were further reduced for birth countries with lower HIV prevalence, possibly reflecting lower likelihoods of clinicians offering testing to those populations. Addressing HIV-associated TB through integrated prevention and care remains a key commitment of the WHO End TB strategy [27].

Higher levels of drug resistance were found among migrants with TB compared with Irish-born, with the level of MDR broadly similar to that previously reported among migrants with TB in Europe [17]. Most patients infected with MDR-TB strains in this study originated in Eastern Europe, reflecting background levels reported in surveillance data for these origin countries [28]. Although advances in MDR-TB treatment have been made, it remains a significant obstacle to TB elimination, particularly as 80% of patients with MDR-TB in this study had a pulmonary component, which was comparable with findings from other international studies [29].

Two key social determinants, housing type and employment status, were significant at univariable level. Migrants with TB had an increased odds of living in congregate residential settings compared with Irish-born patients, which is a key risk factor for transmission [6,30]. Migrants with TB also had higher adjusted odds of being in paid employment compared with Irish-born patients, indicating a potential benefit from directly observed treatment via video rather than in-person to facilitate continued attendance in the workplace and support treatment success [31].

Our study aligned with several origin countries with the highest CIRS reported by Vasiliu et al. and Domaszewska et al. among migrant TB patients [32,33]. Both these studies also reported CIRs that diverged from the published country-specific WHO incidence estimates. Similar to this study, migrants from Eritrea and Somalia had higher CIRs compared with the WHO estimate, whereas migrants from Mongolia, Nepal, and Pakistan migrants had lower CIRs. Higher CIRs among Eritreans in Ireland could be influenced by the majority also being IPARs (75%) and consequently being more likely to experience both adverse conditions and increased exposures during hazardous migration pathways, combined with an increased case-detection rate due to being offered TB screening during voluntary initial health assessments at IPA accommodation centers [34]. Conversely, the lower rates we observed among migrants from India and Mongolia are likely influenced by the fact that migrants from these countries are not routinely offered screening and arrive more often as economic migrants who travel under better conditions. A lower prevalence was estimated for visa applicants from India, Pakistan, and Nepal in Australia and was thought to be a reflection of the type of migrant applying for residence [35].

The findings that recently arrived migrants with TB had higher proportions of IPARs, PTB, and PLWH are likely influenced by the practice of offering initial health assessments, which include pulmonary TB symptom screening and HIV testing, to IPAs upon arrival in Ireland [34]. However, IPARs remain a small proportion of migrants in this study overall (12% of migrants with TB) and include refugees as well as IPAs. A study of migrant TB in low TB incidence European destination countries between 2014 and 2020 also found higher proportions of PTB among recently arrived migrants, which was similarly thought to be linked to the use of chest X-ray-based screening for IPAs [36].

Although it is commonly accepted that the highest risk of TB among migrants occurs in the first few years after arrival in the destination country due to reactivation of remotely acquired infection in the origin country or via new infection acquired during hazardous conditions along the migration pathway [3,5,37], other studies have found that the increased risk persists for several years after arrival and can be influenced by recurring travel to the origin country [[38], [39]]. Similar to a recent study of migrants with TB in Europe, this study found that over half of migrants with TB presented more than 5 years after arrival in Ireland, and that the proportions of migrants with TB from high and very high TB incidence countries remain relatively stable in all intervals examined [36]. Possible explanations for the longer duration between arrival and notification in the Irish context also include the lack of a systematic TB screening program and high levels of extrapulmonary disease, which may be more difficult to diagnose in non-specialist settings, given the wide-ranging clinical presentations and often paucibacillary nature of extrapulmonary specimens combined with reduced sensitivity of nucleic acid amplification tests on non-respiratory samples [40]. The longer intervals indicate a need to ensure continued access to TB diagnosis and care for the migrant population, in addition to active case finding in high-risk populations.

### Strengths

This study utilized a programmatic data source of all patients with TB notified in Ireland, helping to reduce selection bias. Patients were reported according to a standardized European Union case definition, aiding generalizability of the results to other jurisdictions. We analyzed data for all countries of birth, rather than pre-selected based on the highest numbers, to provide an alternate view of TB risk among migrants via CIRs. This study is the first to characterize recent migrants with TB in Ireland and includes several key time periods, before and after the global migration peak in 2015/2016 and the early COVID-19 pandemic period, but prior to recent wars in Ukraine and Palestine, which are currently influencing the epidemiology of TB through increased asylum seeking.

### Limitations

This study was mostly limited by issues relating to the availability of denominator data and poor completeness of the numerator data. Census data may underestimate the true number of migrants resident in Ireland, particularly among undocumented migrants, causing some rates to be overestimated. Conversely, undocumented migrants may be underdiagnosed, leading to an underestimation of rates presented. The 2016 Census of population was used as the 2022 Census was not available for all countries of birth reported among TB patients. It is possible that population changes during the intervening period have reduced the accuracy of CIRs calculated in this study, by either under or overestimating the CIRs, depending on the direction of change in the birth country populations. Population data on recently arrived migrants were not available by country of birth; thus, it was not possible to calculate birth country-specific CIRs for this key population. Key variables such as HIV status and social risk factors had low levels of completeness, which may have introduced bias within the results.

## Conclusions and recommendations

Although screening for PTB remains a key tool in reducing the infectious reservoir, a heightened awareness of EPTB within health systems is needed. Future TBI screening programs will also need to rule out EPTB prior to offering TBI treatment. The pace of TB decline among migrants is slower than among Irish-born and has plateaued in the final years of this study period, making TB elimination targets more difficult to achieve. Elevated CIRs observed among migrant subpopulations indicate that factors in addition to incidence in the birth country need to be considered when evaluating the risk of TB among migrants. Denominator data by country of birth for recently arrived migrants is needed to understand the dynamics of migration in Ireland and how it may be linked to the evolving epidemiology of infectious diseases such as TB. More complete data are needed for key indicators such as HIV status and year of arrival in the country. Differences in the epidemiology of TB reported by this study can be used to inform and enhance future TB service provision and promote migrant health.

## Declaration of competing interest

The authors have no competing interests to declare.

## Appendix Supplementary materials

Image, application 1

## Footnotes

Supplementary material associated with this article can be found, in the online version, at doi:10.1016/j.ijregi.2025.100763.

## Footnotes

1 Council regulation (EU) 2016/679 of the European Parliament.

2 Infectious Diseases Regulations 1981 (S.I. No. 390 of 1981).

3 Data Protection Act 2018 (Section 36(2)) (Health Research Regulations).
