# Supplementary material for: Differences found in patient profiles and incidence trends between migrants and native-born tuberculosis patients in Ireland: A cross-sectional analysis of national surveillance data, 2011-2021
Source: IJID Reg. 2025 Sep 15;17:100763. doi: 10.1016/j.ijregi.2025.100763 (PMC12547294; doi:10.1016/j.ijregi.2025.100763)
Supplement: Supplementary file 1 [file mmc1.docx]

# **Supplementary materials**


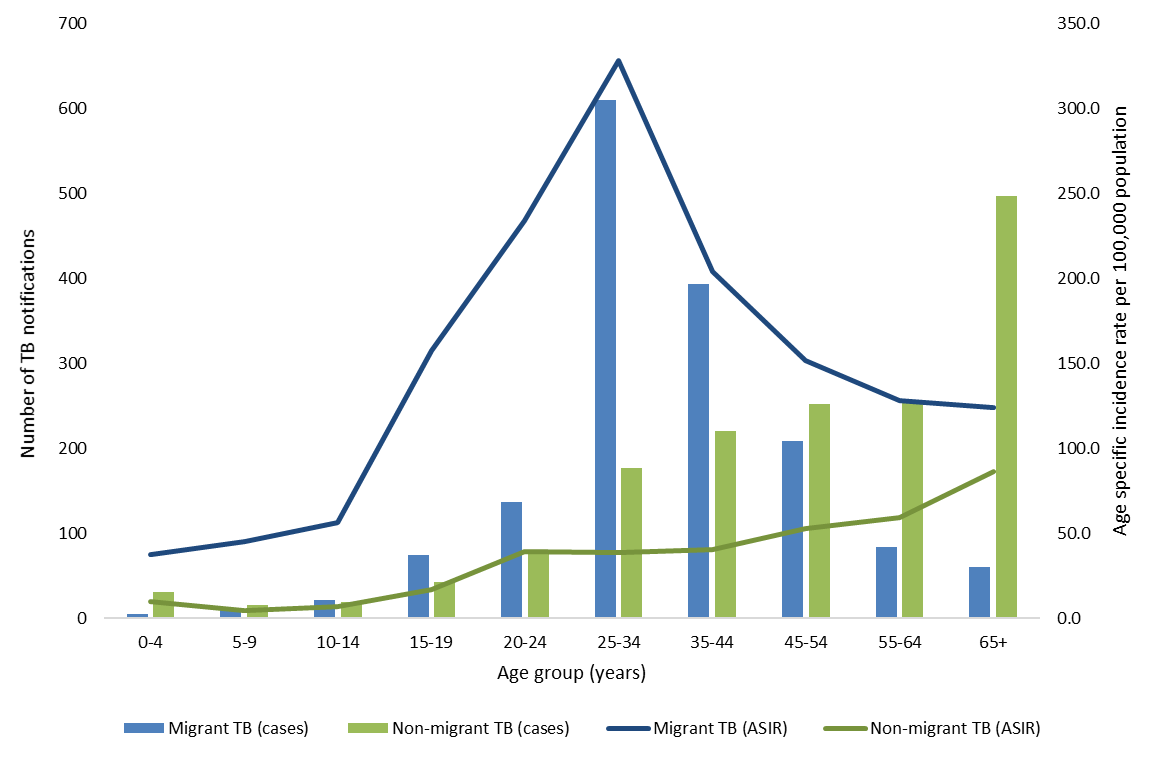


Figure A1. Cumulative number of TB patients and age-specific incidence rate per 100, 000 population by migrant status.


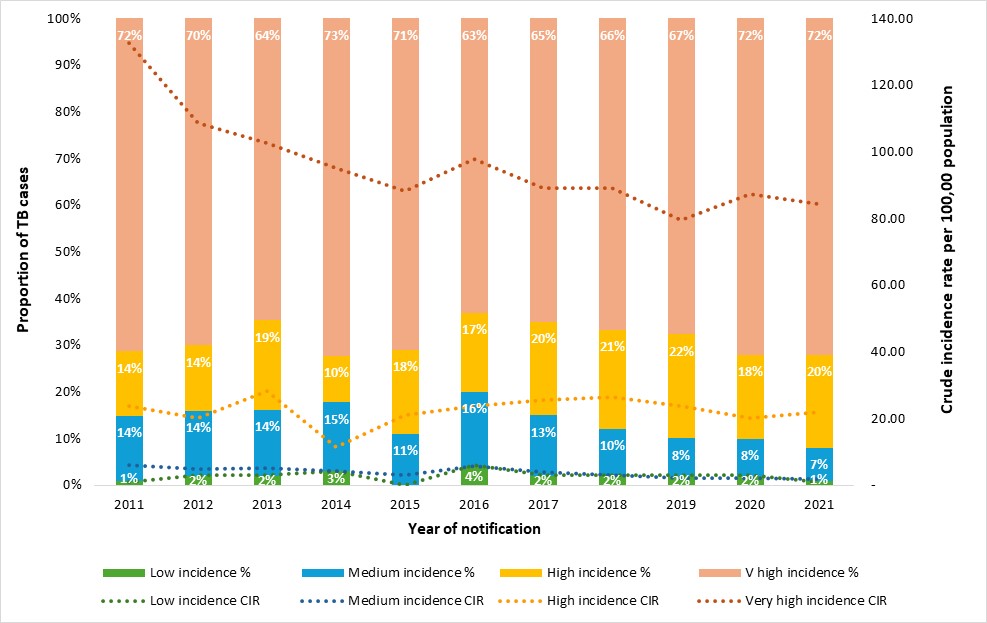


Figure A2. Annual proportion and crude incidence rates by WHO incidence categories for migrants with TB in Ireland.

Table A1. Patient characteristics of migrants with TB by WHO incidence estimate category^[[1]](#footnote-1)^, Ireland.

| **Characteristic** | **WHO TB incidence category** | | | | | **p-value^[[2]](#footnote-2)^** |
| --- | --- | --- | --- | --- | --- | --- |
|  | **Overall**  N = 1,602 | **Low**  N = 30 | **Medium**  N = 193 | **High**  N = 280 | **Very high**  N = 1,099 |  |
| **Median age (years), (IQR)** | 34.0 (16.0) | 43.5 (25.5) | 36.0 (23.3) | 35.0 (16.0) | 33.0 (15.0) | **<0.001** |
| *(% missing)* | 0.1 | 0 | 0.5 | 0 | <0.1 |  |
| **Sex** |  |  |  |  |  | **0.035** |
| *Female* | 687 (43%) | 11 (37%) | 74 (39%) | 104 (37%) | 498 (45%) |  |
| *Male* | 911 (57%) | 19 (63%) | 118 (61%) | 176 (63%) | 598 (55%) |  |
| *(% missing)* | 0.2 | 0 | 0.5 | 0 | 0.3 |  |
| **International Protection Applicant** | |  |  |  |  | **<0.001** |
| *No* | 1088 (88%) | 24 (100%) | 155 (95%) | 194 (93%) | 715 (85%) |  |
| *Yes* | 148 (12%) | 0 (0%) | 8 (4.9%) | 14 (6.7%) | 126 (15%) |  |
| *(% missing)* | 23 | 20 | 16 | 26 | 23 |  |
| **People living with HIV** |  |  |  |  |  | 0.20 |
| *Negative* | 711 (87%) | 13 (93%) | 73 (91%) | 129 (91%) | 496 (85%) |  |
| *Positive* | 106 (13%) | 1 (7.1%) | 7 (8.8%) | 13 (9.2%) | 85 (15%) |  |
| *(% missing)* | 49 | 53 | 59 | 49 | 47 |  |
| **Disease site** |  |  |  |  |  | **<0.001** |
| *Pulmonary* | 916 (58%) | 24 (80%) | 157 (82%) | 204 (73%) | 531 (49%) |  |
| *Extrapulmonary* | 667 (42%) | 6 (20%) | 35 (18%) | 75 (27%) | 551 (51%) |  |
| *(% missing)* | 1.2 | 0 | 0.5 | 0.4 | 1.5 |  |
| **Outbreak associated** |  |  |  |  |  | **0.021** |
| *Not linked to outbreak* | 1,527 (95%) | 27 (90%) | 177 (92%) | 266 (95%) | 1,057 (96%) |  |
| *Outbreak associated* | 75 (4.7%) | 3 (10%) | 16 (8.3%) | 14 (5.0%) | 42 (3.8%) |  |
| *(% missing)* | 0 | 0 | 0 | 0 | 0 |  |
| **Previous TB screening in Ireland** | |  |  |  |  | 0.072 |
| *No* | 1,042 (89%) | 22 (100%) | 136 (90%) | 192 (92%) | 692 (87%) |  |
| *Yes* | 134 (11%) | 0 (0%) | 15 (9.9%) | 17 (8.1%) | 102 (13%) |  |
| *(% missing)* | 27 | 27 | 22 | 25 | 28 |  |
| **Previous TB diagnosis)** |  |  |  |  |  | 0.14 |
| *No* | 1,097 (92%) | 22 (96%) | 136 (93%) | 180 (88%) | 759 (93%) |  |
| *Yes* | 92 (7.7%) | 1 (4.3%) | 11 (7.5%) | 24 (12%) | 56 (6.9%) |  |
| *(% missing)* | 26 | 23 | 24 | 27 | 26 |  |
| **First line drug resistance** | |  |  |  |  | 0.94 |
| *Sensitive* | 1,017 (83%) | 16 (80%) | 125 (84%) | 192 (82%) | 684 (83%) |  |
| *Resistant* | 213 (17%) | 4 (20%) | 24 (16%) | 42 (18%) | 143 (17%) |  |
| *(% missing)* | 23 | 33 | 23 | 16 | 25 |  |
| **M/XDR-TB** |  |  |  |  |  | **0.038** |
| *No* | 1,187 (97%) | 20 (100%) | 141 (95%) | 220 (94%) | 806 (97%) |  |
| *Yes* | 43 (3.5%) | 0 (0%) | 8 (5.4%) | 14 (6.0%) | 21 (2.5%) |  |
| *(% missing)* | 23 | 33 | 23 | 16 | 25 |  |

| **^[[3]](#footnote-3)^Characteristic** | **Missing** | **Overall**  N = 3,198 | **Non-migrant**  N = 1,593 | **Migrant**  N = 1,605 | **Univariable** | | **Multivariable (N=825)** | |
| --- | --- | --- | --- | --- | --- | --- | --- | --- |
|  |  |  |  |  | **cOR** **(95% CI)** | **p-value** | **aOR** **(95% CI)** | **p-value** |
| **Median age (IQR)** | 4 (0.1%) | 40.0 (28.0) | 53.0 (33.0) | 34.0 (16.0) | 0.95 (0.95 to 0.96) | **<0.001** | 0.97 (0.95 to 0.98) | **<0.001** |
| **Sex** | 4 (0.1%) |  |  |  |  | **<0.001** |  |  |
| *Female* |  | 1,276 (40%) | 588 (37%) | 688 (43%) | — |  |  |  |
| *Male* |  | 1,918 (60%) | 1,005 (63%) | 913 (57%) | 0.78 (0.67 to 0.89) |  |  |  |
| **Geographical area** | 0 (0%) |  |  |  |  | **<0.001** |  | **0.037** |
| *East* |  | 1,387 (43%) | 563 (35%) | 824 (51%) | — |  | — |  |
| *Midlands* |  | 155 (4.8%) | 69 (4.3%) | 86 (5.4%) | 0.85 (0.61 to 1.19) |  | 3.26 (1.24 to 9.69) |  |
| *Midwest* |  | 196 (6.1%) | 104 (6.5%) | 92 (5.7%) | 0.60 (0.45 to 0.82) |  | 1.10 (0.63 to 1.92) |  |
| *North East* |  | 228 (7.1%) | 115 (7.2%) | 113 (7.0%) | 0.67 (0.51 to 0.89) |  | 1.45 (0.57 to 4.05) |  |
| *North West* |  | 115 (3.6%) | 77 (4.8%) | 38 (2.4%) | 0.34 (0.22 to 0.50) |  | 0.62 (0.27 to 1.47) |  |
| *South* |  | 610 (19%) | 396 (25%) | 214 (13%) | 0.37 (0.30 to 0.45) |  | 0.65 (0.41 to 1.03) |  |
| *South East* |  | 262 (8.2%) | 146 (9.2%) | 116 (7.2%) | 0.54 (0.42 to 0.71) |  | 0.76 (0.46 to 1.28) |  |
| *West* |  | 245 (7.7%) | 123 (7.7%) | 122 (7.6%) | 0.68 (0.52 to 0.89) |  | 1.16 (0.58 to 2.37) |  |
| **Employment status** | 371 (12%) |  |  |  |  | **<0.001** |  | **0.024** |
| *Paid employment* |  | 1,041 (37%) | 399 (28%) | 642 (45%) | — |  | — |  |
| *Unemployed* |  | 929 (33%) | 428 (30%) | 501 (35%) | 0.73 (0.61 to 0.87) |  | 0.82 (0.56 to 1.19) |  |
| *Retired* |  | 443 (16%) | 403 (29%) | 40 (2.8%) | 0.06 (0.04 to 0.09) |  | 0.27 (0.12 to 0.60) |  |
| *Student/Child* |  | 329 (12%) | 135 (9.6%) | 194 (14%) | 0.89 (0.69 to 1.15) |  | 0.86 (0.45 to 1.70) |  |
| *Other* |  | 85 (3.0%) | 46 (3.3%) | 39 (2.8%) | 0.53 (0.34 to 0.82) |  | 0.81 (0.35 to 1.90) |  |
| **Current housing type** | 343 (11%) |  |  |  |  | **<0.001** |  |  |
| *Private house* |  | 2,646 (93%) | 1,352 (94%) | 1,294 (92%) | — |  |  |  |
| *Congregate residential setting* | | 105 (3.7%) | 36 (2.5%) | 69 (4.9%) | 2.00 (1.34 to 3.05) |  |  |  |
| *Homeless* |  | 27 (0.9%) | 16 (1.1%) | 11 (0.8%) | 0.72 (0.32 to 1.54) |  |  |  |
| *Prison* |  | 22 (0.8%) | 9 (0.6%) | 13 (0.9%) | 1.51 (0.65 to 3.67) |  |  |  |
| *Residential care facility* |  | 20 (0.7%) | 18 (1.2%) | 2 (0.1%) | 0.12 (0.02 to 0.40) |  |  |  |
| *Other housing* |  | 35 (1.2%) | 14 (1.0%) | 21 (1.5%) | 1.57 (0.80 to 3.16) |  |  |  |
| **Disease site** | 36 (1.1%) |  |  |  |  | **<0.001** |  | **<0.001** |
| *Pulmonary* |  | 2,140 (68%) | 1,223 (78%) | 917 (58%) | — |  | — |  |
| *Extrapulmonary* |  | 1,022 (32%) | 353 (22%) | 669 (42%) | 2.53 (2.17 to 2.95) |  | 3.14 (2.09 to 4.79) |  |
| **Outbreak associated** | 0 (0%) |  |  |  |  | **<0.001** |  | **<0.001** |
| *Not linked to outbreak* |  | 2,875 (90%) | 1,345 (84%) | 1,530 (95%) | — |  | — |  |
| *Outbreak associated* |  | 323 (10%) | 248 (16%) | 75 (4.7%) | 0.27 (0.20 to 0.35) |  | 0.16 (0.09 to 0.28) |  |
| **People living with HIV** | 1,804 (56%) |  |  |  |  | **<0.001** |  | **<0.001** |
| *Negative* |  | 1,266 (91%) | 554 (96%) | 712 (87%) | — |  | — |  |
| *Positive* |  | 128 (9.2%) | 22 (3.8%) | 106 (13%) | 3.75 (2.38 to 6.16) |  | 3.80 (1.99 to 7.73) |  |
| **First line drug resistance** | 838 (26%) |  |  |  |  | **<0.001** |  | **0.001** |
| *Sensitive* |  | 2,065 (88%) | 1,046 (93%) | 1,019 (83%) | — |  | — |  |
| *Resistant* |  | 295 (13%) | 82 (7.3%) | 213 (17%) | 2.67 (2.05 to 3.51) |  | 2.30 (1.37 to 4.01) |  |
| **M/XDR-TB** | 838 (26%) |  |  |  |  | **<0.001** |  |  |
| *No* |  | 2,315 (98%) | 1,126 (100%) | 1,189 (97%) | — |  |  |  |
| *Yes* |  | 45 (1.9%) | 2 (0.2%) | 43 (3.5%) | 20.4 (6.26 to 125) |  |  |  |
| **Previous TB screening in Ireland** | 887 (28%) |  |  |  |  | **<0.001** |  |  |
| *No* |  | 1,953 (85%) | 908 (80%) | 1,045 (89%) | — |  |  |  |
| *Yes* |  | 358 (15%) | 224 (20%) | 134 (11%) | 0.52 (0.41 to 0.65) |  |  |  |
| **Previous TB diagnosis** | 801 (25%) |  |  |  |  | **0.086** |  |  |
| *No* |  | 2,188 (91%) | 1,089 (90%) | 1,099 (92%) | — |  |  |  |
| *Yes* |  | 209 (8.7%) | 117 (9.7%) | 92 (7.7%) | 0.78 (0.58 to 1.04) |  |  |  |
| **Patient type** | 362 (11%) |  |  |  |  | **0.004** |  |  |
| *Hospital inpatient* |  | 1,658 (58%) | 876 (61%) | 782 (56%) | — |  |  |  |
| *Hospital outpatient* |  | 914 (32%) | 425 (29%) | 489 (35%) | 1.29 (1.10 to 1.52) |  |  |  |
| *Other* |  | 264 (9.3%) | 145 (10%) | 119 (8.6%) | 0.92 (0.71 to 1.19) |  |  |  |
| **Diabetes** | 1,862 (58%) |  |  |  |  | >0.99 |  |  |
| *No* |  | 1,204 (90%) | 648 (90%) | 556 (90%) | — |  |  |  |
| *Yes* |  | 132 (9.9%) | 71 (9.9%) | 61 (9.9%) | 1.00 (0.70 to 1.43) |  |  |  |
| **Immunosuppression** | 1,841 (58%) |  |  |  |  | **0.033** |  |  |
| *No* |  | 1,067 (79%) | 569 (76%) | 498 (81%) | — |  |  |  |
| *Yes* |  | 290 (21%) | 175 (24%) | 115 (19%) | 0.75 (0.58 to 0.98) |  |  |  |
| **Substance use** | 1,881 (59%) |  |  |  |  | **<0.001** |  |  |
| *No* |  | 945 (72%) | 475 (62%) | 470 (86%) | — |  |  |  |
| *Yes* |  | 372 (28%) | 297 (38%) | 75 (14%) | 0.26 (0.19 to 0.34) |  |  |  |

| **Country of birth** | **Mean CIR** | **Median CIR** | **Mean WHO estimate** | **Ranking in Ireland** | **WHO Mean CIR category** | **Years with patients** | **Total patients** | **% of migrant with TB** |
| --- | --- | --- | --- | --- | --- | --- | --- | --- |
| Eritrea | 443.5 | 609.8 | 110.7 | Top 10 CIR | Very high | 7 | 8 | 0.5 |
| Botswana | 359.7 | 359.7 | 336.3 | Top 10 CIR | Very high | 6 | 11 | 0.7 |
| Somalia | 284.8 | 333.3 | 269.5 | Top 10 CIR & number | Very high | 10 | 47 | 2.9 |
| Malawi | 269.6 | 237.2 | 200.0 | Top 10 CIR | Very high | 8 | 25 | 1.6 |
| Mongolia | 166.2 | 261.1 | 428.0 | Top 10 CIR | Very high | 6 | 7 | 0.4 |
| Indonesia | 161.4 | 0.0 | 325.7 | Top 10 CIR | Very high | 5 | 6 | 0.4 |
| Uganda | 150.9 | 207.5 | 201.7 | Top 10 CIR | Very high | 6 | 8 | 0.5 |
| Nepal | 147.2 | 124.5 | 261.6 | Top 10 CIR | Very high | 8 | 13 | 0.8 |
| India | 137.4 | 133.5 | 244.1 | Top 10 CIR & number | Very high | 11 | 317 | 19.8 |
| Pakistan | 126.9 | 100.8 | 268.1 | Top 10 CIR & number | Very high | 11 | 180 | 11.2 |
| Philippines | 83.3 | 81.5 | 555.6 | Top 10 number | Very high | 11 | 135 | 8.4 |
| South Africa | 73.1 | 61.8 | 858.0 | Top 10 number | Very high | 11 | 65 | 4.0 |
| Nigeria | 36.8 | 30.2 | 219.0 | Top 10 number | Very high | 11 | 67 | 4.2 |
| Romania | 35.2 | 41.8 | 73.8 | Top 10 number | High | 11 | 111 | 6.9 |
| Lithuania | 12.3 | 12.0 | 48.9 | Top 10 number | High | 11 | 45 | 2.8 |
| Poland | 4.7 | 4.3 | 17.2 | Top 10 number | Medium | 11 | 59 | 3.7 |
| United Kingdom | 1.5 | 1.1 | 10.3 | Top 10 number | Medium | 11 | 47 | 2.9 |

| **Characteristic**  N (%) | **Overall**  N = 1,071 | **Interval between arrival in Ireland and TB diagnosis** | | | |  |
| --- | --- | --- | --- | --- | --- | --- |
|  |  | **0-1**  N = 239 | **2-4**  N = 295 | **5-9**  N = 239 | **10+**  N = 298 | ***P*-value^[[4]](#footnote-4)^** |
| **Median age (IQR)** | 34.0 (15.0) | 31.0 (12.0) | 30.0 (11.0) | 34.0 (12.0) | 42.0 (16.0) | **<0.001** |
| *(% missing)* | 0.2 | 0 | 0 | 0.4 | 0.3 |  |
| **Sex** |  |  |  |  |  | 0.33 |
| *Female* | 473 (44%) | 104 (44%) | 120 (41%) | 116 (49%) | 133 (45%) |  |
| *Male* | 596 (56%) | 135 (56%) | 175 (59%) | 123 (51%) | 163 (55%) |  |
| *(% missing)* | 0.2 | 0 | 0 | 0 | 0.7 |  |
| **International Protection Applicant** | |  |  |  |  | **<0.001** |
| *No* | 799 (86%) | 164 (77%) | 223 (85%) | 174 (89%) | 238 (93%) |  |
| *Yes* | 127 (14%) | 49 (23%) | 38 (15%) | 22 (11%) | 18 (7.0%) |  |
| *(% missing)* | 14 | 11 | 12 | 18 | 14 |  |
| **WHO TB incidence category** |  |  |  |  |  | **0.016** |
| *Low* | 20 (1.9%) | 3 (1.3%) | 1 (0.3%) | 6 (2.5%) | 10 (3.4%) |  |
| *Medium* | 110 (10%) | 15 (6.3%) | 23 (7.9%) | 30 (13%) | 42 (14%) |  |
| *High* | 184 (17%) | 49 (21%) | 50 (17%) | 34 (14%) | 51 (17%) |  |
| *Very high* | 749 (70%) | 172 (72%) | 217 (75%) | 169 (71%) | 191 (65%) |  |
| *(% missing)* | 0.7 | 0 | 1.4 | 0 | 1.3 |  |
| **Previous TB screening in Ireland** | |  |  |  |  | **0.006** |
| *No* | 814 (88%) | 202 (94%) | 226 (89%) | 172 (84%) | 214 (85%) |  |
| *Yes* | 113 (12%) | 13 (6.0%) | 29 (11%) | 32 (16%) | 39 (15%) |  |
| *(% missing)* | 13 | 10 | 14 | 15 | 15 |  |
| **Previous TB diagnosis** |  |  |  |  |  | 0.67 |
| *No* | 839 (93%) | 192 (92%) | 225 (93%) | 192 (95%) | 230 (92%) |  |
| *Yes* | 65 (7.2%) | 16 (7.7%) | 17 (7.0%) | 11 (5.4%) | 21 (8.4%) |  |
| *(% missing)* | 16 | 13 | 18 | 15 | 16 |  |
| **Disease site** |  |  |  |  |  | **0.007** |
| *Pulmonary* | 626 (59%) | 163 (68%) | 167 (57%) | 129 (54%) | 167 (56%) |  |
| *Extrapulmonary* | 443 (41%) | 76 (32%) | 127 (43%) | 110 (46%) | 130 (44%) |  |
| *(% missing)* | 0.2 | 0 | 0.3 | 0 | 0.3 |  |
| **Months between onset and diagnosis, Median (IQR)** | 2.0 (3.2) | 1.7 (2.2) | 2.1 (3.0) | 2.0 (3.7) | 2.6 (3.7) | **0.014** |
| *(% missing)* | 32 | 33 | 30 | 33 | 31 |  |
| **Outbreak associated** |  |  |  |  |  | **0.034** |
| *Not linked to outbreak* | 1,026 (96%) | 232 (97%) | 274 (93%) | 232 (97%) | 288 (97%) |  |
| *Outbreak associated* | 45 (4.2%) | 7 (2.9%) | 21 (7.1%) | 7 (2.9%) | 10 (3.4%) |  |
| *(% missing)* | 0 | 0 | 0 | 0 | 0 |  |
| **People living with HIV** |  |  |  |  |  | **0.004** |
| *Negative* | 542 (89%) | 103 (80%) | 154 (91%) | 120 (92%) | 165 (90%) |  |
| *Positive* | 69 (11%) | 26 (20%) | 15 (8.9%) | 10 (7.7%) | 18 (9.8%) |  |
| *(% missing)* | 43 | 46 | 43 | 46 | 39 |  |
| **First line drug resistance** |  |  |  |  |  | 0.59 |
| *Sensitive* | 697 (81%) | 158 (81%) | 196 (79%) | 165 (84%) | 178 (81%) |  |
| *Resistant* | 160 (19%) | 36 (19%) | 52 (21%) | 31 (16%) | 41 (19%) |  |
| *(% missing)* | 20 | 19 | 16 | 18 | 27 |  |
| **M/XDR-TB** |  |  |  |  |  | 0.39 |
| *No* | 826 (96%) | 187 (96%) | 235 (95%) | 191 (97%) | 213 (97%) |  |
| *Yes* | 31 (3.6%) | 7 (3.6%) | 13 (5.2%) | 5 (2.6%) | 6 (2.7%) |  |
| *(% missing)* | 20 | 19 | 16 | 18 | 27 |  |
| **Employment status** |  |  |  |  |  | **<0.001** |
| *Paid employment* | 479 (46%) | 76 (33%) | 136 (47%) | 115 (49%) | 152 (52%) |  |
| *Unemployed* | 365 (35%) | 92 (40%) | 89 (31%) | 81 (35%) | 103 (35%) |  |
| *Retired* | 26 (2.5%) | 9 (3.9%) | 4 (1.4%) | 2 (0.9%) | 11 (3.8%) |  |
| *Student/Child* | 147 (14%) | 44 (19%) | 50 (17%) | 32 (14%) | 21 (7.2%) |  |
| *Other* | 29 (2.8%) | 10 (4.3%) | 10 (3.5%) | 4 (1.7%) | 5 (1.7%) |  |
| *(% missing)* | 2.3 | 3.3 | 2.0 | 2.1 | 2.0 |  |
| **Current housing type** |  |  |  |  |  | **<0.001** |
| *Private house* | 952 (92%) | 183 (79%) | 271 (93%) | 218 (94%) | 280 (98%) |  |
| *Congregate residential / care setting* | 60 (5.8%) | 35 (15%) | 15 (5.2%) | 8 (3.4%) | 2 (0.7%) |  |
| *Prison / Homeless* | 10 (1.0%) | 2 (0.9%) | 2 (0.7%) | 4 (1.7%) | 2 (0.7%) |  |
| *Other housing* | 16 (1.5%) | 11 (4.8%) | 2 (0.7%) | 2 (0.9%) | 1 (0.4%) |  |
| *(% missing)* | 3.1 | 3.3 | 1.7 | 2.9 | 4.4 |  |

1. WHO incidence estimates were not available for two countries, Kosovo (n=2 patients) and Reunion (n=1 patient), in the Irish dataset. [↑](#footnote-ref-1)
2. Kruskal-Wallis rank sum test; Pearson's Chi-squared test; Fisher's exact test [↑](#footnote-ref-2)
3. “Congregate residential setting” includes those reported as being resident in the following types of accommodation at the time of their notification with TB: Bed and Breakfast / Hotel, Hostel, Institution, State Provided Migrant Accommodation. [↑](#footnote-ref-3)
4. Kruskal-Wallis rank sum test; Pearson's chi-squared test. [↑](#footnote-ref-4)
